# Inorganic, organic, and encapsulated minerals in vegetable meal based diets for *Sparus aurata* (Linnaeus, 1758)

**DOI:** 10.7717/peerj.3710

**Published:** 2017-10-27

**Authors:** David Domínguez, Simona Rimoldi, Lidia E. Robaina, Silvia Torrecillas, Genciana Terova, María J. Zamorano, Vasileios Karalazos, Kristin Hamre, Marisol Izquierdo

**Affiliations:** 1 Grupo de Investigación en Acuicultura (GIA), University Institute Ecoaqua, University of Las Palmas de Gran Canaria, Telde, Las Palmas, Canary Islands, Spain; 2 Department of Biotechnology and Life Sciences, University of Insubria, Varese, Italy; 3 Inter-University Centre for Research in Protein Biotechnologies, “The Protein Factory”, Polytechnic University of Milan and University of Insubria, Varese, Italy; 4 BioMar Hellenic SA, Volos, Greece; 5 National Institute of Nutrition and Seafood Research (NIFES), Bergen, Norway

**Keywords:** Inorganic, Organic, Encapsulated, Vegetable meals, Manganese, Selenium, Zinc, *Sparus aurata*, Vertebral morphology

## Abstract

Substituting fishmeal (FM) with vegetable meal (VM) can markedly affect the mineral composition of feeds, and may require additional mineral supplementation. Their bioavailability and optimal supplementation levels depend also on the form of delivery of minerals. The aim of the study was to determine the effect of different delivery forms of three major trace elements (Zn, Mn and Se) in a marine teleost. Gilthead sea bream juveniles of 22.5 g were fed a VM-based diet for 12 weeks that was either not supplemented with these minerals or supplemented with inorganic, organic, or encapsulated inorganic forms of minerals in triplicate and compared to a FM-based diet. Our results showed that mineral delivery form significantly affected the biochemical composition and morphology of posterior vertebrae. Supplementation of VM-based diets with inorganic forms of the target minerals significantly promoted growth, increased the vertebral weight and content of ash and Zn, enhanced bone mineralization and affected the vertebral shape. Conversely, encapsulation of inorganic minerals reduced fish growth and vertebral mineral content, whereas supplementation of organic minerals, enhanced bone osteogenesis by upregulating bone morphogenetic protein 2 (*bmp2)* gene and produced vertebrae with a larger length in relation to height. Furthermore, organic mineral forms of delivery downregulated the expression of oxidative stress related genes, such as Cu/Zn superoxide dismutase (Cu/Zn *sod*) and glutathione peroxidase 1 (*gpx-1*)*,* suggesting thus that dietary minerals supplemented in the organic form could be reasonably considered more effective than the inorganic and encapsulated forms of supply.

## Introduction

Gilthead sea bream (*Sparus aurata*) is one of the main marine finfish produced in the European aquaculture (APROMAR, 2015). Commercial feeds for gilthead sea bream have been traditionally based in fishmeal (FM) and fish oil (FO). These ingredients are limited resources with a tendency to decrease their production (Tacon & Metian, 2008; Tacon & Metian, 2009). Vegetable meals (VM) and oils (VO) can partially replace FM and FO in gilthead sea bream diets (Robaina et al., 1995; Montero et al., 2003; Gómez-Requeni et al., 2004; Izquierdo et al., 2005; Benedito-Palos et al., 2007). However, this replacement may decrease antioxidant status (Saera-Vila et al., 2009). These issues may arise even when diets are formulated to satisfy the recommended nutrient requirements for essential fatty acids or amino acids (NRC, 2011). This suggests possible nutritional imbalances, which can be partly related to differences in the content of zinc (Zn), manganese (Mn), and selenium (Se) between FM and VM (NRC, 2011; Hansen & Hemre, 2013), and can affect bone morphology and antioxidant status.

Zn is involved in bone formation and mineralization by activating osteoblastic cells and inhibiting osteoclastic bone resorption (Yamaguchi, 1998). Zn also forms part of several metalloenzymes that are involved in antioxidant defence such as Cu/Zn-SOD. In Jian carp the activity of Cu/Zn-SOD was increased with increasing dietary Zn levels (Feng et al., 2011). Low dietary Zn may cause slower growth rates in several fish species including Nile tilapia (Do Carmo e Sá et al., 2004), hybrid striped bass (Buentello, Goff & Gatlin, 2009), Jian carp (Tan et al., 2011), grass carp (Liang et al., 2012), and Malabar grouper (Houng-Yung et al., 2014). In rainbow trout low Zn content in the feed may also cause cataracts, skin and fin erosion (Ogino & Yang, 1978), and dwarfism (Satoh et al., 1983). Studies focused on Zn requirement have been carried out on gilthead sea bream, using FM based diets (Serra et al., 1996). FMs contain high concentrations of Zn, whereas VMs are generally low in this mineral (NRC, 2011). For this reason, early studies in gilthead sea bream investigated the effects of increased Zn in diets in which FM was substituted by VM (Robaina et al., 1998).

Mn is a cofactor for metal-enzyme complexes, essential for the antioxidant defence through Mn-SOD. Gibel carp and yellow catfish fed a Mn-deficient diet showed reduced growth (Pan et al., 2008; Tan et al., 2012), whereas cataracts and dwarfism were described in rainbow trout and common and Gibel carps (Ogino & Yang, 1980; Satoh et al., 1983; Yamamoto et al., 1983; Satoh et al., 1992; Pan et al., 2008).

Similarly, Se plays an important role in reducing oxidative stress by being part of the selenoproteins such as glutathione peroxidase 1 *(gpx*-1), whose hepatic activity represent a robust and sensitive criterion to define Se deficiency (Pacitti et al., 2013; Fontagné-Dicharry et al., 2015). Se supplementation in diets for sea bass markedly reduced the occurrence of muscular dystrophy and oxidative risk, enhanced fish growth (Betancor et al., 2012) and adequate skeletal development (Saleh et al., 2014). Bone morphogenetic proteins (*bmp*) and osteocalcin (*oc*) are considered important indicators of bone development and mineralization and are positively related to Se inclusion (Saleh et al., 2014). *Bmp* are involved in a series of cascades that lead to osteoblast differentiation and osteogenesis in fish (Smith et al., 2006). Oc, instead, is an osteoblast-specific gene encoding a secreted protein which represents the most abundant non-collagenous protein of bone matrix (Sommer et al., 1996). This gene is generally inactivated during osteoblast proliferation, while it is abundantly transcribed during osteoblast differentiation. Osteocalcin is released by osteoblasts during bone formation and binds to the mineralized bone matrix (Hauschka & Wians Jr, 1989).

Inorganic minerals may be more effectively absorbed if they are present in their chelated organic forms (Apines et al., 2003; Apines et al., 2004). Some authors have described an increase in Se absorption in fish fed an organic source of Se (Paripatananont & Lovell, 1997). Similarly, several studies have shown that Zn and Mn are absorbed better when fish receive them in inorganic forms than organic or chelated to amino acids (Watanabe, Kiron & Satoh, 1997; Do Carmo e Sá et al., 2005). Other studies have reported higher bioavailability of these minerals when organic compounds are used instead of inorganic ones (Paripatananont & Lovell, 1995; Satoh et al., 2001; Apines et al., 2004; Fountoulaki et al., 2010). Minerals can also be delivered through encapsulation, thus reducing the interactions occurring with other minerals when they are supplied in excess. However, there is a lack of consistency in the repeatability of these results and important variations are found among different authors’ data (Antony Jesu Prabhu, Schrama & Kaushik, 2014).

Production of gilthead sea bream is constrained by the high incidence of skeletal anomalies, commonly reaching up to 100% (as cited in Boglione et al., 2001). Radiological studies serve as a useful method for classifying vertebral anomalies (Boglione et al., 2001; Witten et al., 2009). However, the relationship between vertebral mineral content and bone morphology has not been extensively studied with respect to experimental diets (Roy & Lall, 2003; Poirier Stewart et al., 2014). Many radiological studies are based upon observations, and very few studies have actually used vertebral measurements to accurately describe vertebral morphology and anomalies (Fjelldal, Nordgarden & Hansen, 2007; Poirier Stewart et al., 2014).

Accordingly, the objective of the present research was to further study the effects of Mn, Zn, and Se on bone development and oxidative stress markers by supplementing such minerals in different delivery forms in the gilthead sea bream diets for with low levels of FM and FO.

**Table 1:** Ingredients and analyzed proximate composition of the experimental diets supplemented with different sources of target minerals (Zn, Mn and Se).

Ingredients (%)	C-	DE	DO	DI	FM
Fish meal^a^	15	15	15	15	63
Corn gluten	22	22	22	22	
Soya cake^b^	20	20	20	20	
Soya protein concentrate	10	10	10	10	
Wheat gluten	3.8	3.8	3.8	3.8	
Wheat	11.73	11.43	11.33	11.33	20.52
Fish oil^c^	7.5	7.5	7.5	7.5	8
Rapeseed oil^d^	7.5	7.5	7.5	7.5	8
Microingredients^e^	2.02	2.02	2.02	2.02	0.3
Premix vitamins and minerals^f^	0.45	0.45	0.45	0.45	0.45
Inorganic target minerals^g^				0.4	
Chelated (organic) target minerals^h^			0.4		
Encapsulated (chitosan) target minerals^i^		0.3			
Proximate composition (%)					
Moisture	8.1	8.2	8.1	8.1	7.0
Crude protein	44.2	43.7	43.5	42.5	45.0
Crude lipid	19.6	20.3	19.6	20.8	23.6
Ash	5.7	5.6	5.4	5.8	10.4
Mineral Composition					
Zn (mg kg^−1^)	39	120	150	140	53
Mn (mg kg^−1^)	22	62	52	52	13
Se (mg kg^−1^)	0.55	0.89	1.20	0.90	1.70
Ca (%)	0.95	0.98	0.86	1.00	2.20
P (%)	1.00	1.10	0.98	0.99	1.60
Ca:P	0.95	0.89	0.88	1.01	1.38

**Notes.**

a South-American, Superprime.

b 48 Hi Pro Solvent Extr.

c STD 18.

d European, non-GM, double-low quality rapeseed oil.

e Contains monocalcium phosphate, lysine, methionine and yttrium.

f Contains vitamins and minerals to satisfy known nutritional requirements excluding the target minerals (Zn, Mn and Se) (DSM Nutritional Products, Basel, Switzerland).

g Zinc oxide, manganese oxide and sodium selenite (DSM Nutritional Products, Basel, Switzerland).

h Se Methionine, Zn, and Mn chelated to amino acids (DSM Nutritional Products, Basel, Switzerland).

i Encapsulated (chitosan) zinc oxide, manganese oxide and sodium selenite.

## Material and Methods

The animal experiments described comply with the guidelines of the European Union Council (2010/63/EU) for the use of experimental animals and have been approved by the Bioethical Committee of the University of Las Palmas de Gran Canaria (REF: 007/2012 CEBA ULPGC).

### Diets

In order to determine the effect of supplementing low FM diets with zinc (Zn) manganese (Mn) and selenium (Se) by using different mineral delivery forms, five diets (manufactured by BioMar Tech-Centre, Brande, Denmark) were formulated (Table 1). Specifically, a low-FM, plant based diet (15% FM) was formulated without any supplementation of Zn, Mn and Se (negative control, C-). This basal diet, was then supplemented with the aforementioned target minerals in inorganic (DI, zinc oxide as ZnO (Zn 72%), manganese oxide as MnO_2_ (Mn 60%), and sodium selenite), organic (DO, Se-methionine, Mn- and Zn-amino acids chelated) or inorganic encapsulated (DE). The encapsulated minerals were prepared according to Berthold, Cremer & Kreuter (1996) by SPAROS (Faro, Portugal) by precipitation-coacervation, in which chitosan was solved in a 2% (v/v) acetic acid solution and encapsulated particles were prepared by dropping the target mineral solution containing sodium selenite, manganese oxide and zinc oxide (Table 1). For comparison, a FM- and FO-based diet (FM) without supplementation of the target minerals was also included. For each of the supplemented experimental diets, a premix consisting of the target minerals in the different delivery forms (inorganic, organic and encapsulated) was prepared and added to the basal diet in order to ensure efficient mixing with the rest of the feed ingredients. Mineral composition of each diet was determined (Table 1). All diets were isoenergetic and isonitrogenous and were formulated to meet all known nutritional requirements for rainbow trout (NRC, 2011) including vitamins and minerals apart from the target ones (Table 1).

### Fish and experimental conditions

For the study, 1,725 gilthead sea bream (*Sparus aurata*) juveniles, obtained by natural spawning from our own broodstock (University of Las Palmas de Gran Canaria Las Palmas, Spain), were randomly distributed into fifteen 500-L circular fiberglass tanks at a density of 115 fish/ tank. Initial mean body weight and total length (TL) were 22.5 ± 1.5 g and 117 ± 4 mm (mean ± SD), respectively. Tanks in a flow-through system were supplied with filtered seawater at 22.8–24.3 °C and kept under a natural photoperiod (July-October) of approximately 12 h of light. Water-dissolved oxygen ranged between 6.5 and 6.9 ppm. Each diet was fed to triplicate groups until apparent satiation three times per day for 12 weeks. To monitor growth, individual fish were anesthetized with clove oil (Guinama S.L.U., Valencia, Spain) and weighed after 47 and 84 days of feeding. At the end of the study, five fish per tank were collected for whole body chemical composition; 20 for radiographic study; eight fish per tank for vertebral axis weight, six fish per tank for vertebrae and liver gene expression studies, and three fish per tank for chemical and mineral composition of the vertebrae. Vertebrae gross chemical composition was determined from haemal vertebrae. Total vertebral weight was compared to total fish weight (vertebral weight/fish weight) to avoid the effect of weight differences.

### Biochemical analysis

Chemical composition of diets, and vertebrae was determined by following standard procedures (Association of Official Analytical Chemists (AOAC, 2000). Crude lipid was extracted according to the method of Folch, Lees & Stanley (1957) and ash by combustion in a muffle furnace at 600 °C for 12 h and at 550 °C for vertebrae; protein content (N × 6.25) was determined by using the Kjeldahl method (AOAC, 2000) and dry matter content was determined after drying the sample in an oven at 105 °C until reaching constant weight. Chemical composition of fish was determined using near-infrared spectroscopy (FoodScan, Foss, Sweden). The evaluation of the mineral content was conducted by means of an inductively coupled plasma mass spectrometry (iCAPQ ICP-MS) at a private, certified laboratory (LDG, Barcelona, Spain), after submitting the sample to acid digestion.

### Vertebral morphometry

Radiographs were taken using a fixed X-ray apparatus (Bennett B-OTC, Bennett X-ray Corp., Chicago, IL, USA) and a 35 × 43 cm digital film (Fujifilm FDR D-EVO (Fujifilm Corporation, Tokyo, Japan). Fish were radiographed in groups of ten. Radiographs were treated digitally (Onis 2.4, DigitalCore, Co.Ltd, Tokyo, Japan) and height, length and intervertebral spaces of eight different vertebrae were measured (vertebrae 3–6 and 13–16) (Fjelldal et al., 2006). A series of measurements were used to describe vertebral morphometry, including vertebral height, vertebral length and vertebral surface area. Additionally, vertebral weight and length (mm) were studied in relation to total length (mm) of fish (vertebral weight/fish total length and vertebral length/fish total length) and the vertebral length and height relationship was calculated (vertebral length/vertebral height) to study vertebral shape. All these parameters were measured individually for each of the 20 fish per tank studied and served to define morphometric differences among fish fed the different diets.

### Gene expression

#### RNA extraction

Six fish were sampled from each tank and divided into two pools of three fish. Total RNA was extracted from 60 mg of liver and 150 mg of posterior vertebrae using TRI Reagent^®^ Solution (Life Technologies, Carlsbad, CA, USA) and purified on RNeasy Mini Spin Columns (Qiagen, Hilden, Germany) following the manufacturer’s instructions.

#### Reverse transcription

Reverse transcription of 1 µg total RNA from each experimental sample was performed with the iScript cDNA synthesis kit (Bio-Rad Laboratories, Hercules, CA, USA) according to the manufacturer’s instructions with slight modifications. Briefly, 1 µg total RNA and nuclease-free water to a final volume of 15 µl were heated at 65 °C for 10 min and cooled in ice. Afterwards 1 µl of iScript reverse transcriptase and 4 µl of 5 × iScript reaction mix were added, reaching a final reaction volume of 20 µl. The complete reaction mix was incubated for 5 min at 25 °C, 30 min at 42 °C, and then 5 min at 85 °C to inactivate reverse transcriptase. For gene quantification, the reverse transcription reactions were diluted 1:10.

#### Quantitative PCR

The nucleotide sequences of primers used in this study are reported in Table 2.

**Table 2:** Sequences of primers used for gene expression analysis.

Gene	Symbol	Nucleotide sequence
Alpha-actin	*α*-*act*	F: 5′-TCTGTCTGGATCGGAGGCTC-3′
		R: 5′-AAGCATTTGCGGTGGACG-3′
Ribosomal protein 27a	*rpl-27a*	F: 5′-ACAACTCACTGCCCCACCAT-3′
		R: 5′-CTTGCCTTTGCCCAGAACTT-3′
Cu/Zn superoxide dismutase	*sod*	F: 5′-TTGGAGACCTGGGCAACGTGA-3′
		R: 5′-TCCTGCTTGCCTCCTTTTCCC-3′
Glutathione peroxidase 1	*gpx-1*	F: 5′-GCTTTGAGCCAAAGATCCAG-3′
		R:5′-CTGACGGGACTCCAAATGAT-3′
Bone morphogenetic protein 2	*bmp2*	F: 5′-GTGGCTTCCATCGTATCAACATTTT-3′
		R: 5′-GCTCCCCGCCATGAGT-3′
Osteocalcin	*oc*	F: 5′-AGCCCAAAGCAGGTAAGCAAG-3′
		R: 5′-TTTCATCACGCTACTCTACGG-3′

A total of 2 µl of diluted cDNA was used in real-time PCR for gene expression quantification using IQ™ SYBR Green Supermix (Bio-Rad Laboratories, Hercules, CA, USA)**.** Duplicate analyses were performed for each sample for both the housekeeping and the target gene in a final reaction volume of 20 µl.

*β*-actin and ribosomal protein 27a (*rpl-27a*) were used as housekeeping genes to normalize the expression of oxidative stress genes (*gpx-1*, *sod*) in liver and of osteogenesis genes (*oc*, *bmp2*) in posterior vertebrae, respectively. Real-time quantitative PCR was performed using the iQ5 Multicolor Real-Time PCR detection system (Bio-Rad Laboratories, Hercules, CA, USA). The PCR conditions were as follows: 95 °C for 3 min and 30 sec, followed by 40 cycles of 95 °C for 15 sec, 58.1 °C for 30 sec, and 72 °C for 30 sec; 95 °C for 1 min, and a final denaturation step from 58 to 95 °C for 10 sec. The 2^−ΔΔCt^ method was applied to analyse the relative changes in gene expression.

**Table 3:** Body weight (g) of gilthead sea bream fed diets with different mineral sources at 0, 47, and 84 days.

Body weight (g)	C-	DE	DO	DI	FM
0 days	22.5 ± 0.8	22.6 ± 0.8	22.4 ± 0.8	22.3 ± 0.8	22.5 ± 0.8
47 days	50.0 ± 3.6^a^	50.3 ± 3.6^a^	49.6 ± 3.6^a^	53.0 ± 3.5^b^	55.9 ± 3.6^c^
87 days	81.4 ± 6.1^a^	79.1 ± 6.1^a^	80.8 ± 6.0^a^	84.2 ± 6.1^b^	90.0 ± 6.1^c^

**Notes.**

* Different letters in a row denote significant differences between groups fed different diets for a given feeding period (mean ± SD, *n* = 3, *P* < 0.05).

### Statistics

All data were statistically analysed using STATGRAPHICS Centurion XVI (Version 16.2.04), STATGRAPHICS *plus 5.1* (Statpoint Technologies, Warrenton, VA, USA), or SPSS v21 (IBM Corp., Chicago, IL, USA) and means ± SD were calculated for every parameter measured. Data were tested for normality with the one-sample Kolmogorov–Smirnov test. For normally distributed data, one-way analysis of variance (ANOVA) was used to determine the effects of the different diets. Data were tested for homogeneity and post-hoc analysis was carried out using *Tukey* test if variances were the same or *Games-Howell* test whenever variances were different. Significant differences were considered for *P* < 0.05. When data did not follow a normal distribution, logarithmic or arcsin transformation was carried out or non-parametric tests, such as *Kruskal-Wallis*, were used.

## Results

### Growth

Fish readily accepted experimental diets and no significant differences were found in feed intake between fish fed the different diets. From 47 days of feeding until the end of the trial, body weight was significantly lower in fish fed the VM-based diets, containing only 15% FM and 7.5% FO, than in fish fed the FM diet (Table 3). Whereas supplementation with Zn, Mn and Se in organic (DO) or encapsulated forms (DE) did not affect sea bream growth, addition of the same minerals in inorganic form (DI) significantly improved final body weight (Table 3). Moreover, in fish fed DI diet, the ratio between vertebral weigh and total length ratio resulted significantly higher than in fish fed FM diet (Table 4).

**Table 4:** Vertebral weight/fish total length, vertebral length/fish total length, and vertebral length/vertebral height of gilthead sea bream fed diets with different mineral sources for 12 weeks^*^.

Vertebral Morphometry	C-	DE	DO	DI	FM
Vertebral weight/fish total length (mg mm^−1^)	8.51 ± 2.10^ab^	8.48 ± 2.29^ab^	9.15 ± 1.76^ab^	9.55 ± 2.03^b^	8.24 ± 1.56^a^
Vertebral length/fish total length (mm mm^−1^)^−2^	2.47 ± 0.11^ab^	2.51 ± 0.07^b^	2.49 ± 0.07^ab^	2.47 ± 0.07^a^	2.44 ± 0.09^ab^
Vertebral length/vertebral height (mm mm^−1^)	1.33 ± 0.07^a^	1.39 ± 0.06^c^	1.38 ± 0.07^c^	1.36 ± 0.07^b^	1.33 ± 0.08^a^

**Notes.**

* Different letters in a row indicate significant differences in vertebral weight (mean ± SD, *n* = 3, *P* < 0.05) between experimental groups for the same time period.

### Biochemical analysis

Fish belonging to different feeding groups did not differ in their whole body composition (Table 5). However, DI and DO diets significantly increased the ash content of posterior vertebrae in comparison to fish fed diet C- (Table 6). Feeding with diet DE did not increase ash content, but reduced protein content in the vertebrae compared to C-, DO and DI fed fish (Table 6).

**Table 5:** Initial and final whole body composition (% dry weight) of gilthead sea bream juveniles fed diets with different mineral sources for 12 weeks^*^.

	Initial	C-	DE	DO	DI	FM
Lipid	29.0 ± 0.3	33.3 ± 2.1	34.1 ± 1.9	33.1 ± 0.3	34.6 ± 0.6	33.8 ± 1.2
Ash	11.4 ± 0.7	10.2 ± 0.6	9.5 ± 0.1	9.7 ± 0.1	10.2 ± 0.4	8.9 ± 0.9
Protein	36.6 ± 0.6	36.6 ± 2.0	34.3 ± 2.0	35.1 ± 1.5	33.5 ± 0.4	35.4 ± 1.6
Moisture	67.7 ± 0.9	66.6 ± 0.4	66.2 ± 0.4	66.2 ± 0.6	66.4 ± 0.6	65.6 ± 0.3

**Notes.**

* mean ± SD, *n* = 3.

**Table 6:** Initial and final composition (% dry weight) of posterior vertebrae of gilthead sea bream juveniles fed diets with different mineral sources for 12 weeks^*^.

	Initial	C-	DE	DO	DI	FM
Lipid	28.4 ± 0.9	31.8 ± 1.4	32.1 ± 3.4	32.5 ± 2.0	33.3 ± 0.9	32.0 ± 1.7
Ash	33.0 ± 1.8	32.3 ± 1.8^a^	32.3 ± 1.5^a^	34.2 ± 1.1^b^	34.9 ± 0.9^b^	33.5 ± 1.5^ab^
Protein	33.5 ± 1.8	28.4 ± 0.7^b^	26.9 ± 1.4^a^	29.6 ± 0.9^b^	29.8 ± 1.3^b^	26.5 ± 0.9^a^
Moisture	47.8 ± 1.3	43.6 ± 2.2	44.2 ± 1.2	40.3 ± 7.3	44.3 ± 0.3	43.3 ± 1.0

**Notes.**

* Different letters in a row indicate significant differences (mean ± SD, *n* = 3, *P* < 0.05) between experimental groups for the same time period.

Zn content in vertebra was increased by dietary supplementation of minerals regardless of mineral delivery form (Table 7). Mn and Se content in the vertebrae did not reflect the amount of this mineral in the diet, which was lower in C- and FM diets, since no significant differences were found between the dietary fish groups (Table 7). Finally, among all fish groups, only sea bream fed the DI diet had a Ca and P vertebrae content significantly lower than fish receiving FM diet, although (Table 7). The dietary Ca and P levels did not differ significantly between the VM based diets (Table 1).

**Table 7:** Zn, Mn, Se, Ca, and P content in the posterior vertebrae of gilthead sea bream juveniles fed different mineral sources for 12 weeks^*^.

	Zn (mg kg^−1^)	Mn (mg kg^−1^)	Se (mg kg^−1^)	Ca (%)	P (%)	Ca:P
C-	31.4 ± 1.0^a^	10.9 ± 0.2	0.15 ± 0.01	6.6 ± 0.5^ab^	3.2 ± 0.2^ab^	2.07
DE	34.7 ± 0.2^b^	11.0 ± 0.3	0.15 ± 0.02	6.0 ± 0.5^ab^	2.9 ± 0.3^ab^	2.06
DO	34.9 ± 0.5^b^	11.1 ± 0.5	0.17 ± 0.03	6.3 ± 0.6^ab^	3.0 ± 0.3^ab^	2.07
DI	35.2 ± 1.2^b^	11.2 ± 0.2	0.18 ± 0.03	5.4 ± 0.9^a^	2.7 ± 0.4^a^	2.03
FM	33.4 ± 1.4^ab^	10.7 ± 0.7	0.17 ± 0.03	7.0 ± 0.2^b^	3.4 ± 0.2^b^	2.04

**Notes.**

* Different letters in a row indicate significant differences (mean ± SD, *n* = 3, *P* < 0.05) between experimental groups for the same time period.

**Table 8:** Expression level of osteocalcin (*oc*) and bone morphogenetic protein 2 gene (*bmp2*) in vertebrae of gilthead sea bream juveniles fed diets with different mineral sources for 12 weeks^*^.

Vertebral ossification related genes	C-	DE	DO	DI	FM
*oc*	1.00 ± 0.07^a^	1.94 ± 0.95^ab^	2.04 ± 0.47^ab^	2.25 ± 0.61^ab^	1.59 ± 0.11^b^
*bmp2*	1.10 ± 0.58^a^	3.31 ± 1.93^ab^	7.75 ± 1.00^b^	2.56 ± 0.49^ab^	4.07 ± 1.38^ab^

**Notes.**

* Different letters in a row indicate significant differences in gene expression (mean ± SD, *n* = 6, *P* < 0.05) between experimental groups for the same time period.

### Vertebral morphometry

From our previous studies on vertebral morphology, the posterior vertebra V13 resulted to be more affected by the diet than anterior vertebrae (Supplemental File), and therefore it was used, in this study, to calculate vertebral morphometric parameters. Among the different parameters measured, the ratios between the vertebral length and fish total length and between the vertebral length and vertebral height were significantly affected by dietary treatments (Table 4). In general, inclusion of minerals to a VM-diet did not increase the vertebral length/fish total length ratio in comparison to fish fed diets C- and FM (Table 4). This value was significantly higher in fish fed DE diet compared to those receiving diet DI. Conversely, the shape of the vertebrae was significantly affected by mineral dietary inclusion. Specifically, compared to C- and FM groups, the vertebral length/vertebral height ratio was significantly increased by including encapsulated (DE) and organic (DO) target minerals, and to a lesser extent by inorganic minerals (DI) (Table 4). Only fish fed DI diet showed, instead, a vertebral weight/fish total length value higher than FM group.

### Gene expression

Fish fed diet C- showed the lowest *oc* gene expression in vertebrae, being significantly lower than in fish fed FM diet (Table 8). Mineral supplementation to the VM based diets, improved the transcript levels of *oc,* but the high variation in the data and the small number of samples (*n* = 6) did not allow to show significant differences. Similarly, expression of *bmp2* gene was the lowest in fish fed diet C- and it was increased by adding minerals, being significantly higher in fish fed organic minerals (Table 8). In liver *gpx-1* gene expression tended to be lower, although not significantly, in fish fed organic minerals (DO) as well as FM control diet. Conversely, fish fed encapsulated minerals (DE) showed a significant (*p* < 0.05) upregulation of *sod* gene, whilst those fed C- and FM diets presented the lowest expression. Consequently, fish receiving the diet supplemented with organic or inorganic form of minerals showed intermediate expression level of *sod* (Table 9).

**Table 9:** Expression level for oxidative stress related genes (*gpx-1* and *sod*) in liver of gilthead sea bream juveniles fed diets with different mineral sources for 12 weeks^*^.

Oxidative stress related genes	C-	DE	DO	DI	FM
*gpx-1*	1.01 ± 0.20	1.41 ± 0.59	0.85 ± 0.17	1.38 ± 0.20	0.86 ± 0.15
*sod*	1.00 ± 0.06^c^	4.26 ± 0.14^a^	1.91 ± 0.65^bc^	2.85 ± 0.78^b^	1.35 ± 0.38^c^

**Notes.**

* Different letters in a row indicate significant differences in gene expression (mean ± SD, *n* = 6, *P* < 0.05) between experimental groups for the same time period.

## Discussion

The dietary content of trace elements can affect fish performance and other biomarkers (NRC, 2011; Antony Jesu Prabhu, Schrama & Kaushik, 2014). In the present study, the fish meal based diet was lower in Zn and Mn but higher in Se in comparison to the supplemented diets, whereas the VM-based diet that was without any mineral supplementation had lower content of all three target minerals. Dietary levels of Zn, Mn and Se in the non-supplemented diet (C-) (39, 22 and 0.55 mg/kg, respectively) were lower than the requirements described for marine fish species. For instance, Zn requirements for growth have been reported to be at least 61 mg/kg for sea bream (Carpenè et al., 1999) and 60.2 mg/kg for turbot (*Scophthalmus maximus*) (Ma et al., 2014), whereas Mn requirements are about 25 mg/kg for cobia (*Rachycentron canadon*) (Liu et al., 2013). Se requirements are about 0.8 mg/kg for cobia (Liu et al., 2010), 1.6 mg/kg for grouper *Epinephelus malabaricus* (Lin & Shiau, 2005) and 1.6 mg/kg for juvenile largemouth bass *Micropterus salmoide* (Zhu et al., 2012). The dietary content of three trace elements in our supplemented diets (DI, DO and DE) was instead higher than the requirements described for marine fish species but, anyway, below to levels usually considered to be toxic.

In the present study, fish fed a VM-diet without supplementation of Zn, Mn and Se showed either reduced growth, vertebral length and/or vertebral mineralization of posterior vertebrae, in conjunction with low ash content and the lowest expression of *oc* and *bmp2*, which are biomarkers of bone differentiation and mineralization in gilthead sea bream (Saleh et al., 2014). This could indicate a lack of Zn, Mn or Se, since a deficiency in these minerals has been related to reduced growth, dwarfism and bone malformation (Watanabe, Kiron & Satoh, 1997; Le et al., 2014). Diet containing inorganic trace elements significantly promoted growth in juvenile sea bream in comparison to the other diets. Dietary inorganic Zn has been found to improve growth in other marine and freshwater species, such as grouper (*Epinephelus malabaricus*) (Houng-Yung et al., 2014), yellow catfish (*Pelteobagrus fulvidraco*) (Luo et al., 2011), Jian carp (*Cyprinus carpio var. Jian*) (Tan et al., 2011), and hybrid Tilapia (*Oreochromis niloticus × Oreochromis aureus*) (Zhao et al., 2011). Nevertheless, dietary inorganic Mn could have also contributed to improve growth. Indeed, the increase in Mn has been shown to promote growth in gibel carp (*Carasius auratus gibelio*) (Pan et al., 2008), cobia (*Rachycentron canadon*) (Liu et al., 2013), and yellow catfish (*Pelteobagrus fulvidraco*) (Tan et al., 2012). In particular, in this study, inorganic Zn and Mn were supplemented in the form of oxides (ZnO and MnO_2_, respectively) and selenium was added as sodium selenite (Na_2_SeO_3_). The latter has been used in several previous trials and is considered an effective inorganic source of selenium to fish (Antony Jesu Prabhu, Schrama & Kaushik, 2014). In most previous studies in fish, the inorganic forms of Zn and Mn that are commonly used for dietary supplementation were sulphates (ZnSO_4_ and MnSO_4_, respectively). In general, sulphates have been shown to have higher bioavailability than oxides in fish (NRC, 2011; Antony Jesu Prabhu, Schrama & Kaushik, 2014) and other animals (EFSA Journal, 2012). However, dietary supplementation with ZnO showed no difference to ZnSO_4_ on growth performance, feed utilization and bone Zn concentration and retention in tilapia (Do Carmo e Sá et al., 2005). Moreover, in this species, both Zn inorganic forms (ZnO and ZnSO_4_) showed better results as compared to the amino acid chelated form. In sea bass, dietary supplementation with ZnO showed no significant difference to organic Zn in growth and feed utilization but had lower tissue bioavailability in skin and liver in comparisonto the respective concentration of the organic zinc (Fountoulaki et al., 2010). Conversely, in carp, dietary Mn showed higher availability in the sulphate and chloride forms than in the oxide and carbonate ones (Satoh, Takeuchi & Watanabe, 1987). To our knowledge, this is the first study to evaluate the ZnO and MnO as mineral sources in sea bream diets in a comparative way to organic and encapsulated forms. In line with previous evidences, our findings indicate that the efficiency of different inorganic mineral sources could be species dependent. Nevertheless, in this case, the positive results obtained by the inorganic forms used (i.e., oxides and salts) are promising mineral sources to be used in the supplementation of feeds for sea bream.

Mineral content in the vertebrae has been suggested as the main criterion for estimation of nutritional requirements for Zn and Mn, since fish mainly store these elements in the vertebrae (Antony Jesu Prabhu, Schrama & Kaushik, 2014). The significantly lower Zn content in vertebrae of gilthead sea bream fed the diet non-supplemented with the target minerals might indicate a mineral deficiency; therefore, it means that gilthead sea bream juveniles have a Zn requirement higher than the 39 mg/kg ensured by the not supplemented diet, as previously recommended (Serra et al., 1996). Although Se concentration in cobia vertebrae increased with increasing dietary Se (Liu et al., 2010), Mn and Se content in the vertebrae of our fish did not vary significantly with mineral supplementation though. Lower vertebral Ca and P were found in fish fed inorganic minerals, whereas in the same fish group, vertebral weight and ash, protein and Zn content, increased. Zn has been found to stimulate bone formation through proliferation of osteoblastic cells (Yamaguchi, 1998), however rats fed with excessive levels of Zn (>2,500 mg/kg) showed reduced bone Ca and P in the study of Stewart & Magee (1964). Detailed data of Zn requirements in sea bream is needed; therefore, further dose–response studies are being conducted by our group to understand the effect of organic and inorganic Zn in fish diets with high plant protein inclusion.

Feeding inorganic minerals enhanced the expression of *oc* that is a molecular marker of bone mineralization, and affected vertebral morphology, by increasing their length in relation to height as compared to the group fed with non-supplemented diet. The good correlation between dietary Zn levels and *oc* expression is in agreement with the promotion of bone mineralization by this mineral. In rainbow trout, Zn supplementation at 60 mg/kg levels caused a statistically significant increase of serum alkaline phosphatase (ALP) activity and significantly decreased oxidative stress (Kucukbay et al., 2006). Serum ALP is of particular interest since an increase of its activity is usually associated to osteoblast hyperactivity and bone remodeling and, in human, ALP expression is one of the most frequently used markers of the osteoblast differentiation process (Huang et al., 2013).

The response of oxidative stress related genes *gpx-1* and *sod* to different dietary mineral delivery forms was characterized by a decrease of their expression associated with mineral supplementation in organic delivery forms (DO). In particular, the supplementation of VM-based diet with organic minerals tended to downregulate the expression of *gpx-1* and significantly decreased *sod* transcription levels in liver of gilthead sea bream. Our results are in agreement with the downregulation of *gpx-1* gene expression found in gilthead sea bream larvae fed increased dietary organic Se levels, (Saleh et al., 2014). Se plays an important role in fish antioxidant defenses being a cofactor of the antioxidant enzyme GPX (Felton, Landolt & Grace, 1996). In sea bass larvae, an increase in dietary organic Se downregulated both *gpx-1* and *sod* with a consequent reduction of free radicals production (Betancor et al., 2012). *Sod* expression was reduced by dietary Se supplementation also in *Brycon cephalus* exposed to an oxidative stress-producing agent (Monteiro, Rantin & Kalinin, 2009). However, if enzymatic activity is considered, the effect of Se is the opposite. Indeed, previous studies have reported that dietary supplementation of Se enhanced the antioxidant enzyme capacity in common carp (*Cyprinus carpio*) (Elia et al., 2011), rainbow trout (*Oncorhynchus mykiss*) (Küçükbay et al., 2009), cod (*Gadus morhua*) (Penglase et al., 2010) and yellowtail kingfish (*Seriola lalandi*) (Ilham & Fotedar, 2016). Based on *gpx-1* and *sod* expression data obtained in our study, organic Se seemed to be the most efficient delivery form. This assumption is in agreement with several evidences suggesting that organic Se at higher concentrations is more bioavailable and tolerated than inorganic Se (Paripatananont & Lovell, 1997; Wang et al., 2007; Küçükbay et al., 2009; Rider et al., 2009; Rider et al., 2010; Thiry et al., 2012).

However, this was not entirely true for Zn and Mn. Indeed, gilthead sea bream fed minerals as inorganic oxides showed ash, Zn, and Mn vertebral content comparable to fish fed with organic minerals, thus denoting their high availability. In line with our result, in rainbow trout and hybrid striped bass, proteinate or amino acid chelated Zn did not increase Zn deposition in bone in comparison to inorganic form (Zn sulfate) (Rider et al., 2010; Savolainen & Gatlin, 2010). Nevertheless, in other fish species, higher absorption and retention of Zn and Mn were found when the minerals were supplemented in the organic form (chelated to amino acids or Zn propionate) than in the inorganic form (sulfate or oxide forms) (Spears, 1989; Wedekind, Hortin & Baker, 1992; Hahn & Baker, 1993; Paripatananont & Lovell, 1997; Satoh et al., 2001; Apines et al., 2001). Therefore, it is important to carefully consider in which form minerals are supplemented: in species in which supplementation in organic form may increase mineral availability, it is necessary to reduce their inclusion levels, thus avoiding a possible negative effect due to excess of accumulation. For instance, daily doses of 9–12 mg Zn kg^−1^ body weight d^−1^ resulted to be toxic in carp (*Cyprinus carpio)*, Nile tilapia (*Oreochromis niloticus)*, and guppy (*Poecilia reticulate)* (Clearwater, Farag & Meyer, 2002). Conversely, dietary concentrations of Zn >900 mg kg^−1^ dry diet were relatively nontoxic to several fish species (Clearwater, Farag & Meyer, 2002). Further studies are being conducted to better understand the effects of inorganic and organic Zn and Mn on growth of sea bream.

Integration of VM-based diets with encapsulated minerals did not improve fish growth and led to a higher expression of *sod* genes in liver, suggesting an increase in the oxidative risk. In addition, Zn, Mn, and Se levels in vertebrae tended to be lower in fish fed encapsulated minerals, regardless of their dietary levels. The low protein content in fish fed encapsulated minerals (similar to those fed the FM-based diet) could also be related to a lower Zn availability in its encapsulated form since this mineral has been found to increase the protein component of bone and to promote bone growth via insulin like growth factor 1 action (Ma & Yamaguchi, 2001a; Ma & Yamaguchi, 2001b).

In conclusion, the results of this study showed that supplementation of inorganic Zn and Mn is required in VM-based diets to promote growth in gilthead sea bream. Organic minerals, particularly Se, seemed more effective in reducing oxidative risk, whereas encapsulated delivery forms reduced the ash content of the vertebrae, or in other words, mineral deposition, thus negatively affecting fish growth.

##  Supplemental Information

10.7717/peerj.3710/supp-1 Supplemental Information 1 Vertebral morphology of Gilthead sea bream juveniles Vertebrae 3–6 and 13–16 were measured for length, height and vertebral length/vertebral height. Measurements were carried for fish fed a diet based on vegetable ingredients (C-) without mineral supplementation and fish fed a diet based on fish meal and fish oil. Vertebra 13 was the only vertebra to be different between both treatments for vertebra length and vertebral length/vertebral height.

## Additional Information and Declarations

### Data Availability

The following information was supplied regarding data availability: The raw data is included in the tables in the manuscript.
